# EBF1, PAX5, and MYC: regulation on B cell development and association with hematologic neoplasms

**DOI:** 10.3389/fimmu.2024.1320689

**Published:** 2024-01-22

**Authors:** Li Li, Daiquan Zhang, Xinmei Cao

**Affiliations:** ^1^ Immune Mechanism and Therapy of Major Diseases of Luzhou Key Laboratory, School of Basic Medical Sciences, Southwest Medical University, Luzhou, China; ^2^ Department of Traditional Chinese Medicine, West China Second University Hospital, Sichuan University, Chengdu, China

**Keywords:** EBF1, PAX5, MYC, V(D)J rearrangement, B cell development, hematologic neoplasms

## Abstract

During lymphocyte development, a diverse repertoire of lymphocyte antigen receptors is produced to battle against pathogens, which is the basis of adaptive immunity. The diversity of the lymphocyte antigen receptors arises primarily from recombination-activated gene (RAG) protein-mediated V(D)J rearrangement in early lymphocytes. Furthermore, transcription factors (TFs), such as early B cell factor 1 (EBF1), paired box gene 5 (PAX5), and proto-oncogene myelocytomatosis oncogene (MYC), play critical roles in regulating recombination and maintaining normal B cell development. Therefore, the aberrant expression of these TFs may lead to hematologic neoplasms.

## 1 Introduction

In early lymphocytes, RAG1/2-mediated V(D)J rearrangement of antigen receptor variable region genes results in the diversity of mammalian B cell receptors (BCRs) and T cell receptors (TCRs), which is the base of the adaptive immune response. In the process of recombination, RAG1/2 recognizes and scissors recombination signal sequences (RSSs) that flank the *V*, *D*, and *J* gene segments to generate double-strand breaks (DSBs) (1–4). Then, the broken DNA is repaired by non-homologous end joining (NHEJ) (5).

**Figure 1:**
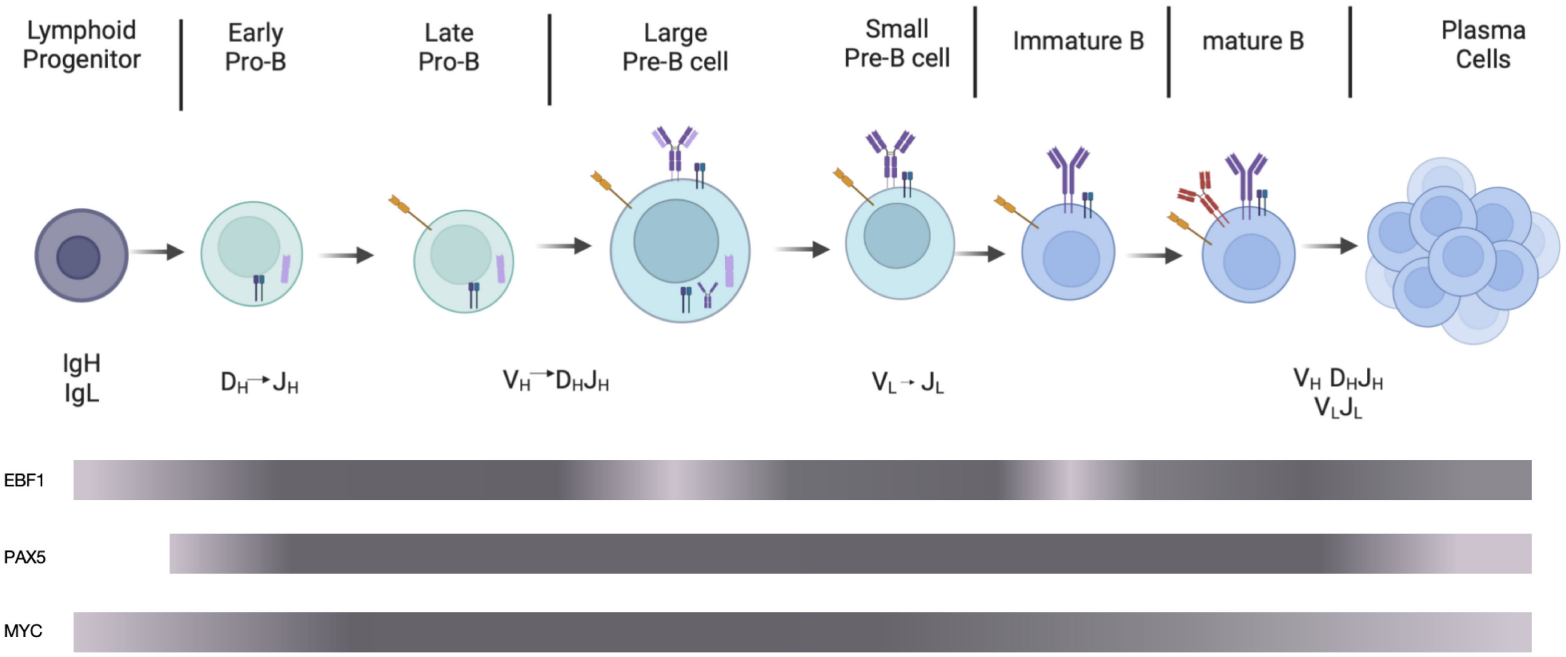
Schematic diagram of B cell development. In the bone marrow, partial lymphoid progenitors differentiate into early pro-B cells performing *D_H_* to *J_H_* rearrangement, followed by late pro-B cells in which *V_H_* to *D_H_J_H_* rearrangement occurs. After large pre-B cells expressing pre-BCR composed of heavy chains, VpreB1, and λ5, small pre-B cells perform light chain rearrangement. Immature B cells, preceding maturation in the periphery, express mature BCRs composed of heavy and light chains. Activated by antigens, mature B cells differentiate into plasma cells, which produce antibodies. The grey bars represent the expression levels of EBF1, PAX5, and MYC. The darker the color is, the higher the protein expression level is. The image was generated using the online platform https://www.biorender.com.

In the bone marrow (BM), B cells are derived from pluripotent stem cells that sequentially differentiate into lymphoid progenitors, progenitor-B (pro-B, including early and late pro-B), precursor-B (pre-B, including large and small pre-B), and immature B cells migrating to the spleen to further differentiate into mature B cells. Additionally, the V(D)J rearrangement of BCR heavy and light chain genes occurs at different stages (6–8). Following light chain gene (*V_L_* to *J_L_*) recombination at the small pre-B stage, heavy chain gene *D* to *J* first rearranges at the early pro-B stage, followed by *V* to *DJ* rearrangement at the late pro-B stage (9) (**Figure 1**).

Accumulative evidence has proven that V(D)J rearrangement in B cells is regulated by various TFs, such as PU.1, Ikaros, E2A, EBF1, PAX5, and MYC (10–14). Forming complex networks, these TFs orderly activate B lineage-specific genes (15), ensuring normal lymphocyte development and avoiding B-cell malignancy (4). This review mainly focuses on the effects of EBF1, PAX5, and MYC on B cell development and their association with hematologic neoplasms.

## 2 EBF1

EBF1 consists of four domains, including the conserved N-terminal DNA binding, the Ig/plexins/TF-like (IPT), the helix-loop-helix, and the C-terminal domain (CTD) (16). So far, only IPT and CTD domain functions have been studied in depth. The EBF1 CTD domain was found to bind to inaccessible genomic regions prior to detection of chromatin accessibility and recruit Brahma-related gene 1 to stabilize its binding to these regions (17, 18). Since a prion-like structure in the CTD domain promotes liquid-liquid phase separation via interaction with RNA-binding protein FUS, EBF1 enhances chromatin opening and facilitates the transition from lymphoid progenitor chromatin to B-lineage one (16, 18, 19). Similarly, Boller et al. found that the EBF1 CTD domain induces CpG demethylation to promote chromatin accessibility and, therefore, determines B cell commitment by regulating specific gene expression (17). The EBF1 IPT domain has been reported to bind to transporter protein 3 to facilitate nuclear import of EBF1 (20).

During stepwise development of B cells, the expression of EBF1 protein varies dynamically. EBF1 protein expression level reaches the highest at the heavy and light chain recombination stage, reaches moderate levels during cytokine-mediated proliferation phase (such as pre-BCR independent proliferation phase at large pre-B cell stage) and lowest at the pre-BCR and BCR signal transduction stage (21) (**Figure 1**). At the common lymphoid precursor (CLP) stage, E2A and FOXO1 activate the *EBF1* gene and enhance its expression. In turn, EBF1 promotes FOXO1 protein expression (21–23). FOXO1 enhances RAG protein expression via direct binding to the *RAG* locus throughout early B cell development (24). At the highly-proliferating early B cell stage, increased EBF1 represses FOXO1 protein expression and its association with the *RAG* locus, negatively regulating RAG protein expression (25, 26). Barberi et al. also found that TF C/EBPɑ upregulates EBF1 protein expression at the CLP stage (27).

In previous studies, EBF1 has been identified to play critical roles in B cell development (28, 29). It was demonstrated that EBF1 is necessary not only for the survival, proliferation, and signaling of pro-B cells and peripheral B-cell subsets (such as B1 cells and marginal zone B cells) but also for germinal center formation and class switch recombination (30). Ikaros and PU.1 contribute to lymphoid progenitor cell differentiation into pro-B cells, and this process is blocked when these two TFs are deficient (31–34). The overexpression of EBF1 induces Ikaros-KO Lin-Sca1^hi^c-Kit^hi^ cells to differentiate into pro-B cells (35). Further studies showed that EBF1 and E2A form a complex that binds to the cis-acting element of the *Igh* locus and activates *D_H_*-*J_H_* rearrangement (36, 37). Like *E2A* knockout mice, *EBF1*-deficient mice show blocked *D_H_*-*J_H_* rearrangement and arrested B-cell development (38). Using the diffuse large B cell lymphoma KIS-1 cell line, Bullerwell et al. found that exogenous expression of EBF1 in KIS-1 cells promote the expression of B cell-specific genes, such as *CD19* and *CD79b*, partly because of enabling PAX5 to interact with mixed lineage leukemia H3K4 methyltransferase complexes (39). The results suggested that EBF1 is able to work synergistically with PAX5 to regulate B cell-specific gene expression. When the EBF1 N-terminus fused with the JAK2 C-terminus to form EBF1-JAK2 (E-J), the transcriptional activity of PAX5 was suppressed, which depends on PAX5 interaction with the fusion protein and JAK2-induced phosphorylation. But the detailed mechanism of inhibition remains elusive (40). The fusion protein was only found in 0.6% of adult BCR-ABL1-negative B-acute lymphoblastic leukemia (ALL) (41). Thus, these findings demonstrate that EBF1 plays essential roles in B cell-specific gene expression and B cell differentiation.

## 3 PAX5

PAX5, also known as B cell specific activating protein (BSAP), contains the N-terminal paired domain responsible for binding DNA, C-terminal regulatory module controlling transcription, and middle conserved octapeptide and partial homeodomain (42–44).

PAX5 is restrictedly expressed in the B-cell lineage in the hematopoietic system, particularly high at the pro-B to mature B-cell stage (45–47) (**Figure 1**). It’s been identified that EBF1 binds directly to the *PAX5* promoter to positively regulate its expression, which IL7-activated-STAT5 may improve at the early B cell stages (48–50). In addition, PU.1, interferon regulatory factor 4 (IRF4; functions as a transcriptional repressor), IRF8, and NF-kB are also important regulators of *PAX5* (51). For example, activation of pre-BCR-induced-class IA phosphoinositide-3 kinase (PI3K) signaling promotes FOXO1 degradation, which upregulates PAX5 protein expression via repressing IRF4 protein expression (52).

It’s well-known that PAX5 plays essential roles in regulating B cell development and activating B cell-specific signaling proteins, such as CD19 and B-cell linker protein (BLNK), which determines the commitment and differentiation of B cells (52). In *PAX5*-deficient mice, B cells are severely blocked at the pro-B stage (47). Furthermore, *PAX5*-deleted mature B cells purified from the lymph nodes of *CD19-cre PAX5^fl/–^ Eμ-BCL2* mice dedifferentiated into functional T cells after transplantation into *RAG2* KO mice (53).

How does PAX5 play regulatory roles in B cell development? David et al. found that in *PAX5*-deficient mice, the rearrangement of distal rather than proximal genes of *Igh V* is significantly impaired in pro-B cells (54). When PAX5 is reintroduced in *PAX5* KO pro-B cells, large-scale contraction of *Igh* loci and rearrangement of distal *V_H_-D_H_J_H_* are induced, suggesting that PAX5 is essential for distal *V_H_* gene rearrangement in pro-B cells (55). Further studies revealed that due to chromatin activation and antisense transcription induction at the *PAX5*-activated intergenic repeat (PAIR) elements in the distal *V_H_* gene cluster, PAX5 promotes *V_H_* to *D_H_J_H_* recombination (56).

Histone modification may regulate the rearrangement of *Igh V* genes. For example, trimethylation of Lys-27 on histone H3 (H3K27me3) and dimethylation of Lys-36 on histone H3 (H3K36me2) is associated with repressive and active genes, respectively (57). Xu et al. also used CHIP and CHIP-on-CHIP techniques to find that H3K36me2 is enriched on distal *V_H_* genes, while H3K27me3 is exclusively on proximal *V_H_* genes. Via cooperation with IL-7 that promotes H3K36me2, PAX5 induces H3K27me3 to balance the proximal and distal *V_H_* gene recombination (58).

Recent studies also showed that cohesion complex–mediated chromatin loop extrusion results in *Igh* locus contraction that is helpful for RAG scanning (59–62). Recruiting polycomb-repressive complex 2 (PRC2), PAX5 downregulates the expression of Wings apart-like protein homolog (WAPL) that releases cohesion from chromatin and inhibits *Igh* loop extraction, enhancing *V_H_* gene rearrangement (59, 63, 64).

Moreover, Zhang et al. found that not only does PAX5 bind to the coding regions of *V_H_* gene segments, but it also interacts with the RAG1/2 complex to promote *V_H_*-to-*D_H_J_H_* recombination (65, 66).

Taken together, these studies demonstrate that Pax5 is crucial to *V_H_* (especial distal *V_H_*) to *D_H_J_H_* recombination and B cell commitment.

## 4 MYC

MYC, as a proto-oncogene, belongs to the basic region/helix-loop-helix/leucine zipper (bHLHZip) transcription factor family, containing the N-terminal transactivating domain (TAD) and the C-terminal DNA-binding domain. The TAD domain is an intrinsically disordered region responsible for the transcriptional activation of MYC. Dependent on the C-terminal domain with bHLHZip fragment, MYC forms a heterodimer with Max, enabling it to bind to DNA (67, 68). In addition to acting as a global gene expression amplifier, MYC regulates cell proliferation, differentiation, apoptosis, and homeostasis (69–72). Notably, MYC mutations or overexpression occur in many tumors (73–75).

In an early study, pre-B cells were found to express MYC in response to IL-7 stimulation, and splenic B cells expressed it due to lipopolysaccharide (LPS) stimulation (76). Moreover, MYC protein is continuously expressed from hematopoietic stem cells to lymphocytes, reaching highest in proliferating B cells, especially at the pro- to pre-B cell stage, and its expression decreases at the mature B cell stage (77, 78) (**Figure 1**).

Accumulated evidence showed that the coordinated action of factors regulates MYC expression. *MYC* is one of the EBF1-directly-targeted genes, and EBF1 promotes its expression in pro-B cells; in lack of EBF1 in pro-B cells, over-expression of PAX5 results in decreased MYC expression, which suggests EBF1 and PAX5 may play opposite roles in regulating MYC expression (79). However, Ramamoorthy et al. identified the *EBF1* ^+/−^ *PAX5* ^+/−^ mouse model for B-ALL and found that MYC is upregulated (80). The results imply that EBF1 and PAX5 may cooperate to limit MYC expression, partially avoiding transformation to B-ALL. Besides, Beer et al. found that EBF1 interacts with Epstein-Barr virus (EBV) nuclear antigen 2 (EBNA2) to stimulate MYC expression in human adenoid primary B cells (81). In pre-B cells, pre-BCR signaling-induced Ikaros and Aiolos are reported to negatively regulate MYC expression via binding its promoter (82, 83).

It was reported that deletion of MYC affects B cell development (84, 85). *N*-*myc^fl/fl^ c-myc^fl/fl^ CD19cre^+^* mice exhibit blockage at the pro- to pre-B cell stage; MYC can elevate Ca^2+^ level in early B cells and activate genes targeted by MYC and Ca^2+^, thereby promoting the proliferation and differentiation of early B cells (86). Additionally, MYC has been reported to regulate cell cycle via activating cyclin-dependent protein kinase 1 (CDK1) to phosphorylate p27 (87), inducing cyclin D1 and D2, and sequestrating p27 and p21 (88). When MYC is suppressed in pre-B cells, the cell proliferation is inhibited due to induction of p27 and downregulation of cyclin D3 (83).

Additionally, MYC plays essential roles in further developing B cells in the germinal center (GC) where immune response occurs (89). Studies showed that in the GC response, B-cell lymphoma 6 (BCL6) is a direct inhibitor of MYC; most GC cells express either BCL6 or MYC (90). In the dark region of the GC, antigenic stimulation activates naïve B cells to express MYC and repress BCL6 transcription, leading to clonal expansion (91). After entering the light zone of GC, about 10% of B cells simultaneously express MYC and BCL6 to promote the cells to enter cell cycles (77, 91, 92).

In general, these studies suggest MYC is able to regulate not only the proliferation and differentiation of early B-cells but also B cell development in GC.

## 5 Hematologic neoplasms resulted from abnormal expression of EBF1, PAX5, and MYC

Since EBF1, PAX5, and MYC are essential for B cell development, their ectopic expression or mutants may lead to abnormal B cell development and hematologic neoplasms, such as B-lymphoid leukemia and lymphoma (93–100).

Accumulative evidence showed that *EBF1* deletion results in the pathogenesis, drug resistance, and relapse of B-progenitor ALL (101, 102). In addition, *EBF1* fused with platelet-derived growth factor receptor beta (*PDGFRB*) has been reported in Ph-like ALL. The fused gene contributes to EBF1 function deficiency and differentiation block (103–105). Moreover, a recent study showed that EBF1 is necessary for propagating mantle cell lymphoma, a lethal mature B cell lymphoma (106).

Depending on advanced assays such as whole genome sequencing, whole transcriptome sequencing, and single nucleotide polymorphism array, *PAX5* alterations were identified in human B-ALL. These alterations mainly include rearrangements with partner genes (such as structural proteins, TFs, and signal transducers), mutation (such as R38H, P80R, and G183S), deletions (usually affecting one allele), and intragenic amplification (such as amplification of exons 2 to 5) (93, 102, 107–114). In addition to B-ALL, *PAX5* mutations and rearrangements (such as *PAX5* t (9, 14) (p13;q32)) were identified in Burkitt’s lymphoma, follicular lymphoma as well as diffuse large B cell lymphoma (DLBCL) (115–122).

Unlike other proto-oncogenes possibly activated by genetic mutations, aberrant MYC expression in transformed cells is caused by chromosomal translocation, gene amplification, and aberrant regulation of its expression (77). In B lymphoblastic leukemia with t (9, 22) *BCR*-*ABL1*, t (v;11) *MLL*, and t (12, 21) *ETV6*/*RUNX1* rearrangement, triggered *MYC* overexpression was more common, while the frequency of *MYC* gene translocation and mutation was low (123–131). In contrast, *Ig*/*MYC* rearrangements induce MYC overexpression in B cell lymphomas, such as Burkitt’s lymphoma and plasmablastic lymphomas (132–134). In double-hit lymphoma, besides *BCL6* rearrangement, *MYC* translocation was also observed (135, 136).

In short, as pivotal TFs in B cell development, the abnormalities of EBF1, PAX5, and MYC result in hematologic tumors.

## 6 Discussion

The development of B cells is a complex process that is comprehensively regulated by multiple factors, such as TF EBF1, PAX5, and MYC. EBF1 is involved in the whole process of B cell development. At the early pro-B cell stage, EBF1 promotes the rearrangement of *D_H_* to *J_H_*. When *D_H_J_H_* recombination succeeds, EBF1 targets PAX5 to activate its expression and subsequently blocks the activation of non-B-lineage genes. PAX5 is critical to *V_H_-D_H_J_H_* recombination, especially distal *V_H_* gene rearrangement. Besides, EBF1 cooperates with PAX5 to regulate MYC expression, which may balance the proliferation and differentiation of early B cells. Therefore, EBF1, PAX5, and MYC lesions may drive B-cell malignancy and induce B-cell lymphomas.

In recent years, advances have been made in the regulatory mechanisms of EBF1, PAX5, and MYC in B cell development and maturation because of the application of DNA loop extrusion and phase separation, which still need in-depth research. Employing advanced test methods such as single-cell sequencing, more regulators and/or targeted genes of these TFs may be identified, which is helpful to optimize the regulatory mechanism of B cell development and maturation. Given that the aberrant expression and function of EBF1, PAX5, and MYC contribute to hematologic neoplasms, therapies targeting them may shed light on these diseases.

## Author contributions

LL: Writing – original draft. DZ: Writing – review & editing. XC: Writing – review & editing.
